# A spatial stream-network approach assists in managing the remnant genetic diversity of riparian forests

**DOI:** 10.1038/s41598-019-43132-7

**Published:** 2019-05-01

**Authors:** Patricia María Rodríguez-González, Cristina García, António Albuquerque, Tiago Monteiro-Henriques, Carla Faria, Joana B. Guimarães, Diogo Mendonça, Fernanda Simões, Maria Teresa Ferreira, Ana Mendes, José Matos, Maria Helena Almeida

**Affiliations:** 1 Centro de Estudos Florestais, Instituto Superior de Agronomia, Universidade de Lisboa, Edifício Azevedo Gomes, Tapada da Ajuda, 1349-017 Lisboa, Portugal; 2 Institute of Integrative Biology, Department of Evolution, Ecology, and Behaviour, University of Liverpool, Crown Street, Liverpool, L69 7ZB United Kingdom; 3 Plant Biology, CIBIO/InBio, Centro de Investigação em Biodiversidade e Recursos Genéticos, Laboratório Associado, Universidade do Porto, Campus Agrário de Vairão, 4485-661 Vairão, Portugal; 4 Ecofield, Monitorizações, Estudos e Projectos, LDA, Carcavelos, Portugal; 5 Centro de Investigação e Tecnologias Agroambientais e Biológicas, Universidade de Trás-os-Montes e Alto Douro, Quinta de Prados, Apartado 1013, 5000-801 Vila Real, Portugal; 6 INIAV - Instituto Nacional de Investigação Agrária e Veterinária, I.P., Unidade Estratégica de Biotecnologia e Recursos Genéticos, Lisboa, Portugal; 7 LabOr- Laboratório de Ornitologia, ICAAM - Instituto de Ciências Agrárias e Ambientais Mediterrânicas, Universidade de Évora, 7002-554 Évora, Portugal; 8 Centre for Ecology, Evolution and Environmental Changes - cE3c, Faculdade de Ciências, Universidade de Lisboa, Lisboa, 1749-016 Portugal

**Keywords:** Riparian ecology, Population genetics, Conservation biology, Ecological modelling

## Abstract

Quantifying the genetic diversity of riparian trees is essential to understand their chances to survive hydroclimatic alterations and to maintain their role as foundation species modulating fluvial ecosystem processes. However, the application of suitable models that account for the specific dendritic structure of hydrographic networks is still incipient in the literature. We investigate the roles of ecological and spatial factors in driving the genetic diversity of *Salix salviifolia*, an Iberian endemic riparian tree, across the species latitudinal range. We applied spatial stream-network models that aptly integrate dendritic features (topology, directionality) to quantify the impacts of multiple scale factors in determining genetic diversity. Based on the *drift hypothesis*, we expect that genetic diversity accumulates downstream in riparian ecosystems, but life history traits (e.g. dispersal patterns) and abiotic or anthropogenic factors (e.g. drought events or hydrological alteration) might alter expected patterns. Hydrological factors explained the downstream accumulation of genetic diversity at the intermediate scale that was likely mediated by hydrochory. The models also suggested upstream gene flow within basins that likely occurred through anemophilous and entomophilous pollen and seed dispersal. Higher thermicity and summer drought were related to higher population inbreeding and individual homozygosity, respectively, suggesting that increased aridity might disrupt the connectivity and mating patterns among and within riparian populations.

## Introduction

Riparian trees are foundation species that support biodiversity and modulate key ecosystem functions through their interactions with flooding and sediment regimes in river channels and their floodplains^1,2^. Rivers have been exposed to long-lasting human pressures worldwide, and they are threatened by climate change and a resurgence of damming plans in response to freshwater and energy demands^3^. On the one hand, river regulation alters peak flows and creates physical barriers to gene flow, which hinder the regeneration dynamics of riverine plant communities^4^. On the other hand, climate-driven changes, such as precipitation shifts, might decouple seed development and dispersal from the discharge regime to which they evolved^5^. Improving our capacity to understand and anticipate changes in these fragile ecosystems is of the utmost importance if we are to mitigate the expected pervasive environmental and societal consequences of hydroclimatic alterations^3^. However, the current approaches used to monitor functional responses of riparian forests to global change do not fully accommodate the dendritic structures of hydrographic networks, which hinders the accurate management of these threatened ecosystems. Here, we combined spatial stream-network (SSN) models with landscape genetics tools to quantify the role of ecological factors in determining the amount and distribution of genetic diversity harboured in riparian forests. By doing so, we tested hypotheses on the main drivers of gene flow and connectivity at the population, basin and regional scales, and we showed that this monitoring tool is suitable to design science-based conservation plans.

Riparian forests are confined along dendritic hydrographic networks with typically directional water flow that disperses seeds and vegetative propagules downstream. Any attempt to investigate riparian genetic patterns requires accommodating the dendritic structure of hydrographic networks and its specific topology, connectivity and directionality within the hierarchical organization of riparian landscapes^6^. Spatial models previously applied to investigate genetic patterns across dendritic structures, such as rivers, have provided interesting but limited information because (1) Euclidean geographic distances disregard the complexities of hydrographic networks within nested watersheds^7^; (2) proxies of the basin hierarchy (i.e., stream order) poorly capture the topological properties of dendritic networks^8^; and (3) models do not tackle the joint effects of environmental and spatial components together, providing an incomplete picture of the multiple factors that drive genetic patterns^9^. SSN models^7^ provide a timely opportunity to investigate the joint effects of environmental drivers and spatial properties on a hydrographic network in riparian forests, and these models provide a complete and validated methodological toolbox^10,11^. These models integrate a set of explanatory variables into a single geostatistical model that can accommodate different spatial autocorrelation structures and use spatial weights to capture the influence of branching, flow direction, and discharge^7^. SSN models have been successfully applied to detect spatial patterns of water chemistry^12^ and the distribution of vagile organisms^7,10,13^, but they have not yet been applied to investigate the distribution of the genetic diversity of riparian forest species across different hydrographic networks.

In this study, we combined landscape genetic tools and SSN models to quantify the impacts of key ecological drivers on the distribution of the genetic diversity and structure of the riparian tree *Salix salviifolia* Brot. at various spatial scales. *S. salviifolia* is a foundation species that preferentially grows in intermediate to large order streams where natural flow, erosion and fluvial sedimentation processes create in-channel deposit bars^14^. In this species, gene flow occurs through pollen grains (transported by wind and insects), seeds (mobilized by wind and water) and vegetative propagules transported by water flow. Therefore, the disruption of the natural water flow and sediment regimes by river regulation threatens the dispersal ability and the genetic connectivity of these populations^15,16^. Furthermore, this taxon is endemic to the Western Iberian Peninsula, listed in the EU Habitats Directive, and it creates habitats that host IUCN Endangered Mediterranean fish species such as *Anaecypris hispanica*. The distribution of *S. salviifolia* encompasses a pronounced climatic gradient that spans from the southern Mediterranean edge to the Temperate ecoregion^17^. Hence, *S. salviifolia* is an ideal model species for studying the environmental and spatial drivers of genetic patterns in riparian species.

**Figure 1:**
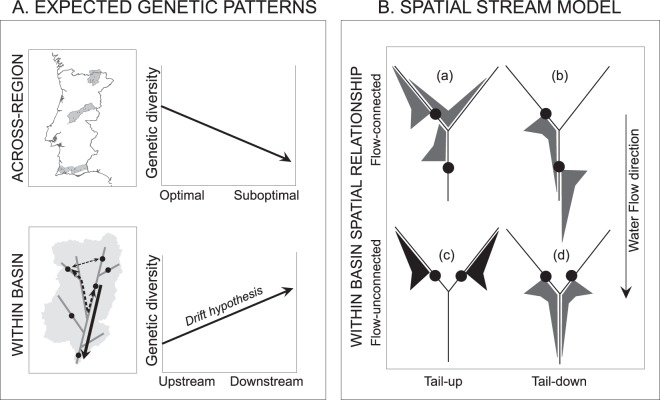
(**A**) Hypothesis tested at different spatial scales for the main drivers of genetic patterns in *Salix salviifolia* populations. At the across-region scale, we expected higher genetic diversity in optimal climatic conditions. At the within-basin scale, we expected asymmetrical dispersal (“*drift hypothesis*”) resulting in the downstream increase in genetic diversity. (**B**) Within-basin spatial relationships (flow-connected/flow-unconnected) of the spatial stream-network model functions (tail-up/tail-down) adapted from Peterson & Ver Hoef^7^. The moving-average functions (MAF) for the tail-up (a, c) and tail-down (b, d) relationships are shown with varying widths representing the strength of the influence for each potential neighbouring site. Spatial autocorrelation occurs between sites when the MAF overlaps (grey), otherwise no spatial autocorrelation is considered (black). The black dots represent sites within the dendritic network.

**Figure 2:**
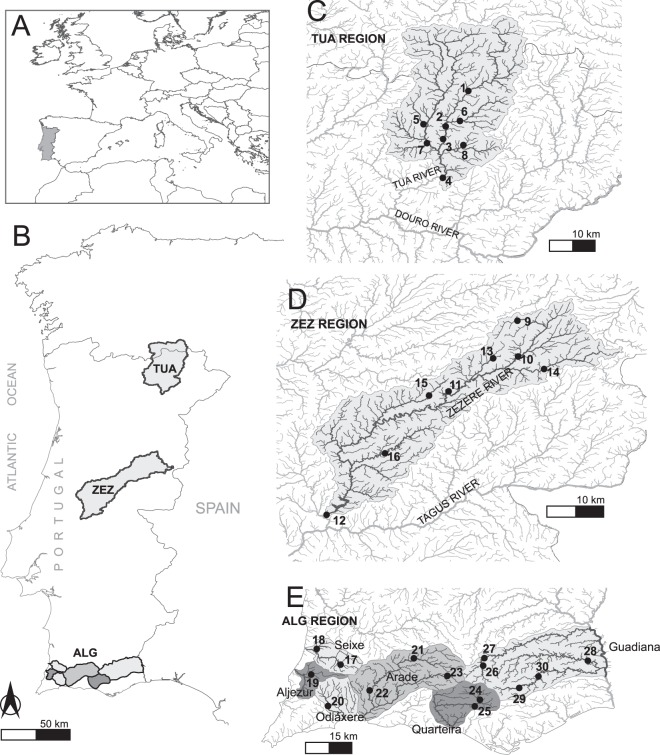
Study area within Europe (**A**), the three studied regions of Tua, Zêzere and Algarve (**B**) and sampled populations within hydrographic networks (**C**–**E**).

Water flow is the main vector that mobilizes propagules for riparian species downstream^18^, which causes the accumulation of riparian plant propagules downstream unless there are other means of upstream dispersal (the so-called *drift-paradox* hypothesis (Fig. 1)^19^. As a result, genetic diversity is expected to increase downstream. This observation has received empirical support that shows a dominant downstream gene flow direction^6,20^, although some studies have documented bidirectional gene flow^9,21^. Dominant winds or foraging preferences by pollinators can move pollen grains and genes upstream^22^. Moreover, local and regional landscape features, such as elevation or climatic gradients, might affect the gene flow patterns within or among basins (Fig. 2), and these features could potentially erase the expected genetic patterns derived from dominant downstream dispersal^8^. Applying SSN models enables us to dissect the contribution of environmental and spatial factors in determining the levels of genetic diversity across basins, populations and individuals. Specifically, this multiscale approach allows us to (i) investigate the genetic diversity levels of riparian populations in basins along an environmental gradient; (ii) quantify the relative contributions of ecological and spatial factors; and (iii) assess the spatial extent at which dominant factors impact the genetic patterns across dendritic networks. Finally, we further discussed the potential applications of SSN models to monitor the responses of riparian ecosystems to global change drivers.

## Results

### Spatial genetic patterns in *Salix salviifolia*

Locally, *ca*. 15% of sites show signs of a deficit or excess of heterozygotes (Table 1), indicating that the allele frequencies at these sites depart from the Hardy-Weinberg Equilibrium (HWE). All estimators of genetic differentiation detected a significant genetic structure within regions (Supplementary Table S1), and the southern region showed the highest genetic structure levels and high proportions of private alleles (PA) (Supplementary Table S2).

**Table 1:** Summary of genetic diversity estimators per population.

Population	Region	n	A_*e*_	uH_*e*_	H_*o*_	F_*is*_	P_1_	P_2_
1	TUA	18	4.4	0.74	0.71	0.026	ns	⋅
2	TUA	54	5.5	0.79	0.79	−0.016	ns	ns
3	TUA	18	5.6	0.73	0.74	−0.003	ns	ns
4	TUA	52	5.4	0.76	0.71	**0.058**	ns	***
5	TUA	18	5.5	0.75	0.71	**0.062**	ns	***
6	TUA	20	4.4	0.76	0.75	−0.007	ns	ns
7	TUA	19	4.0	0.72	0.74	−0.053	ns	ns
8	TUA	15	3.3	0.67	0.81	**−0.252**	***	ns
9	ZEZ	22	4.6	0.75	0.78	**−0.063**	ns	*
10	ZEZ	54	5.7	0.78	0.83	**−0.085**	*	ns
11	ZEZ	18	4.6	0.77	0.78	−0.059	ns	ns
12	ZEZ	55	4.5	0.76	0.82	**−0.094**	**	ns
13	ZEZ	15	4.6	0.73	0.75	−0.073	ns	ns
14	ZEZ	18	5.1	0.78	0.79	−0.046	ns	ns
15	ZEZ	15	4.7	0.78	0.74	0.012	ns	⋅
16	ZEZ	15	3.6	0.67	0.68	−0.065	ns	ns
17	ALG	6	3.7	0.73	0.68	0.034	ns	ns
18	ALG	6	3.8	0.74	0.65	**0.019**	ns	**
19	ALG	30	4.1	0.71	0.74	−0.029	ns	ns
20	ALG	10	5.2	0.79	0.57	**0.260**	ns	***
21	ALG	10	4.1	0.77	0.82	−0.121	ns	ns
22	ALG	10	3.8	0.72	0.67	**−0.002**	ns	**
23	ALG	20	4.3	0.74	0.74	0.021	ns	ns
24	ALG	10	4.1	0.71	0.67	0.009	ns	ns
25	ALG	26	4.1	0.66	0.65	0.061	ns	ns
26	ALG	10	4.3	0.76	0.75	−0.002	ns	ns
27	ALG	14	3.9	0.74	0.85	**−0.216**	**	ns
28	ALG	10	3.9	0.72	0.60	**0.139**	ns	**
29	ALG	8	3.4	0.71	0.67	−0.021	ns	ns
30	ALG	9	2.9	0.62	0.70	−0.135	⋅	ns

Genetic diversity estimated per population as the mean effective allelic richness (*A*_*e*_), expected unbiased heterozygosity (u*H*_*e*_), observed heterozygosity (*H*_*o*_), and inbreeding coefficient (*F*_*is*_). F_is_ values significantly different from zero at p < 0.05 are highlighted in bold. The p-values indicate the significance level of a Hardy-Weinberg test to test for heterozygotes excess (P_1_) and heterozygote deficit (P_2_) across all loci (ns, non-significant; ⋅p-value < 0.1; *p-value < 0.05; **p-value < 0.01; ***p-value < 0.001).

### Geostatistical modelling: covariates and covariance structures

The optimal sets of covariates were similar across the population-level response variables, and the hydrologic index (DA) was selected across the four estimators A_e_, F_is_, uH_e_ and H_o_ (A_e_, number of effective alleles; F_is_, inbreeding; uHe, expected unbiased heterozygosity; H_o_, observed heterozygosity) (Table 2) and had a positive impact on genetic diversity (in terms of A_e_). Therefore, larger and wetter drainage basins tended to correlate with increased levels of genetic diversity. In addition, H_o_ was significantly and positively correlated with altitude (ALT). Climatic covariates were retained for F_is_ and HL (homozygosity level). The thermicity index (BIOC.TH) had a positive effect on F_is_ and, at the individual-level, the summer ombrothermic index (BIOC.SO) significantly affected the HL, with higher HL levels at decreasing BIOC.SO values. This result entails that locations undergoing intense summer droughts tend to host individuals and populations with increased levels of homozygosity.

**Table 2:** Final set of covariates (DA-hydrologic index; ALT-altitude, BIOC.SO-summer ombrothermic index; BIOC.TH-thermicity index) and covariance structures for the best models retained for each response variable at the population (A_e_, number of effective alleles; F_is_, inbreeding; uHe, expected unbiased heterozygosity; H_o_, observed heterozygosity) and at the individual level (HL, homozygosity level).

Dependent variable		Fixed effects parameters				Covariance parameters		
Level	Y	Environmental Factor	Estimate	Std Error	p-value	Name	varcomp	range(m)
Population	A_e_	DA	0.4768	0.1733	0.0105	Spherical.TU	0.4790	8287.01
		ALT	0.0010	0.0008	0.2138	LinearSill.TD	0.2377	154617.24
		R^2^ = 0.2337				Nugget	0.0496	
Population	F_is_	BIOC.TH	0.0002	0.0001	0.0341	Mariah.TU	0.1304	14505.31
		DA	−0.0086	0.0056	0.1336	LinearSil.TD	0.5663	116814.96
						Exponential.EUC	0.0016	1855642.4
		R^2^ = 0.1956				Nugget	0.106	
Population	uHe	DA	0.0152	0.0111	0.1810	Exponential.TU	0.2016	209.89
						Exponential.TD	0.0001	88792.04
						Gaussian.EUC	0.6454	17820.05
		R^2^ = 0.0629				Nugget	0.0901	
Population	H_o_	ALT	0.0002	0.0001	0.0061	LinearSill.TD	0.7025	7257.30
		DA	0.0280	0.0175	0.1211	Exponential.EUC	0.0000	3594.72
		R^2^ = 0.2970				Nugget	0.0004	
Individual	HL	BIOC.SO	−0.0023	0.0008	0.0039	Exponential.TU	0.7537	0.00
						LinearSill.TD	0.1416	11.87
		R^2^ = 0.0137				Nugget	0.0908	

The R^2^ values indicate the percentage of variation explained by environmental factors, while varcomp indicates the percentage of variation explained by each covariance structure within the final model mixture, and the nugget (i.e. the unexplained variation) that accounts for the variability that occurs at a scale finer than the closest measurements, as well as measurement error. The range represents the distance after which the spatial autocorrelation becomes zero.

**Figure 3:**
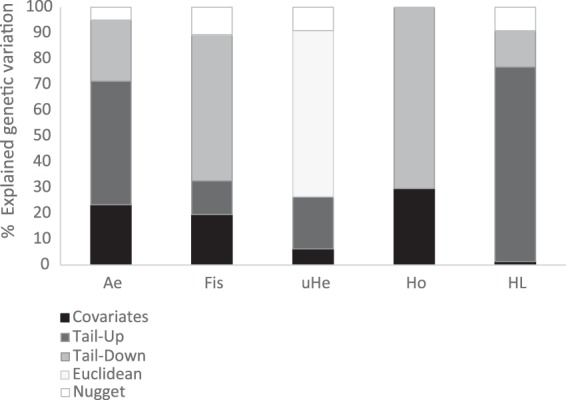
Percentage of genetic variation explained by covariates (environmental factors) and by the different covariance structures within the final model mixture for the analysed genetic estimators at the population (Ae, number of effective alleles; Fis fixation index; uHe (unbiased expected heterozygosity; Ho, observed heterozygosity) and the individual (HL, homozygosity level) level. The nugget represents the unexplained variation.

The optimal covariance structure was a mixture of the possible structures, but with an overriding dendritic model (tail-up or tail-down) across most genetic estimators (Table 2, Fig. 3). The dominant covariance structures were the tail-up for A_e_ and HL, (47.9% and 75.4% explained variation, respectively); the tail-down for F_is_ and H_o_ (56.6% and 70.3% explained variation, respectively) and the Euclidean for uH_e_ (64.5% of the variation explained).

### Geostatistical modelling: Multiscale drivers of genetic diversity

Overall, the retained covariates together explained between 6 and 30% of the genetic variation at the population level for all genetic metrics with a strong hydrologic and climatic component (Table 2, Fig. 3). The percentage of residual variation corresponding to the covariance functions (tail-up, tail-down) accounts for most of the remaining variation. The nugget (variability that cannot be explained by the distance between observations, i.e. the unexplained variation) represented a low (≤10%) percentage of the variation. For all population-level estimators (A_e_, F_is_, uH_e_, H_o_), autocovariance models captured intermediate-scale spatial patterns with spatial ranges of 0.2–20 km. For A_e_ and F_is_, the covariance mixture also captured large-scale patterns of variation (ranges >100 km) (Table 2).

At the individual level, most of the residual variation corresponds to dendritic structures that fitted a tail-up model with a range close to 0 m in the best model for the HL (Table 2, Fig. 3). This result indicates that nearby individuals do not show increased HLs compared to individuals randomly drawn from distant locations in the population. In addition, the tail-down covariance structure captures 14.2% of the variation, and it detected a fine-scale structure (range = 11.9 m). The nugget represented 9.1% of the variability.

## Discussion

The amount of genetic diversity and its spatial distribution within and among populations reflect complex interactions between intrinsic and extrinsic factors such as phenology, dispersal, topography and flood regime^2^. Lately, numerous landscape models have been pursued to disentangle the effects of different ecological and spatial factors in determining the distribution of the genetic diversity across complex landscapes^23^; however, these models fail to capture dendritic structures that characterize riparian habitats. SSN models proved to be suitable for quantifying the impact of environmental factors on shaping spatial genetic patterns, the spatial scale they operate, and their dominant direction (upstream, downstream or both).

The Iberian endemic *Salix salviifolia* displayed higher levels of genetic diversity (uH_e_) compared to other *Salix* species^15,20^, which is coherent with mating processes that favour outcrossing and suggests a relatively good genetic status in this species, at least under the current environmental conditions. Migration history might have modulated the current genetic patterns of these populations, as favourable microclimatic conditions within protected river valleys in the Iberian Peninsula offered refugia for tree species during glaciations^24^. The prevalence of private alleles may be interpreted as genetic evidence for persistence of *S. salviifolia* in the southern part of the peninsula during the last glacial period, as shown for other riparian species^24^.

The movements of propagules or individuals that inhabit riparian habitats are confined to the river flow, either upstream, downstream or both. Our results showed that a large proportion of the residual genetic variation is spatially structured within the basin (i.e., once the effects of ecological factors have been removed). Furthermore, by applying SSN models, we evidenced that the dendritic spatial structure accounted for majority (70–89%) of the variation observed within basins for most estimators of genetic diversity. Therefore, overlooking the dendritic structure of riparian habitats could lead to a misunderstanding of the main ecological drivers that underlie the biodiversity patterns within basins.

The distribution of the genetic diversity across populations is strongly determined by dispersal and gene flow patterns^18^. In *Salix*, the potential sources of gene flow are the movement of pollen grains, seeds and vegetative propagules. Seeds are dispersed by wind (upstream, downstream) and water (mainly downstream with some possible upstream events caused by massive flooding)^4^. The dendritic structure of a basin imposes that the volume of water that flows through the river channel (i.e., river discharge) increases downstream. Our models showed a positive correlation between river discharge and the genetic diversity level that also tended to increase downstream. In addition, the tail-up model explained significant proportions of A_e_ and uH_e_ spatial variation. Both results suggest an important role of hydrochory in mobilizing propagules, as expected based on the *drift* hypothesis^4^. However, our models also noted a significant role of upstream processes that counteracted the dominant downstream movement. For example, the tail-down explained meaningful proportions of A_e,_ F_is_ and H_o_, and the Euclidean model explained large proportion of uH_e_ spatial variation. Upstream seed dispersal has been identified in other riparian species associated with zoochorous and human dispersal^22^. *Salix* spp. are wind and insect pollinated, and these vectors are reported to generate strong genetic patterns that follow dominant wind directions. In river valleys, topography and wind channelling constrain prevailing winds through the hydrographic network^25^, which is a phenomenon that can permit upstream gene movement, as reported in other Salicaceae species^21^. Despite the ability of Salicaceae to resprout from vegetative propagules^26^, the reduced presence of clones in our study discards the notion that they significantly contribute to natural *S. salviifolia* regeneration.

The population genetic diversity estimators (A_e_, F_is_, uH_e_, H_o_) displayed patterns of spatial autocorrelation mostly at the intermediate scale (i.e., 1–100 km *sensu* Fausch^27^) through flow-connected (tail-up) and flow-unconnected (tail-down) spatial relationships (Fig. 1, Table 2). The ranges of these spatial autocorrelation structures suggest that genetic connectivity among *S. salviifolia* populations mainly occurs at the intermediate scale (<20 km), which is likely associated with the interaction of the *Salix* life history with the formation and distribution of hydrogeomorphological landforms (i.e., channel sediment deposits) where the species recruit and colonize^2,28^. Indeed, key biological and physical processes in riparian systems, such as metapopulation dynamics and disturbance regimes, are thought to operate at intermediate scales^27^.

Interestingly, A_e_ showed large-scale spatial autocorrelation (range = 154.6 km) that included flow-unconnected relationships among populations. This result suggests that within hydrographic networks, even remote populations would eventually become connected, possibly integrating a long-term effect of successive pollen and seed dispersal events or through rare long-distance pollen-mediated dispersal events^4^, as in other Salicaceae species^21^.

The HL revealed a limited influence of distance among individuals where only 14.2% of the residual variation in the HL exhibited fine-scale patterns of autocorrelation (range = 11.9 m). The fine-scale spatial aggregation may be primarily due to spatially structured variables, such as microenvironmental heterogeneity. Indeed, fine-scale soil-moisture gradients within riparian habitats are critical for the survival of *Salix* seedlings when the water level decreases after natural flooding events^29^. This finding is consistent with the significant correlation of summer drought with the HL, suggesting that increased aridity may constrain gene flow within basins as drought events become more extreme.

Spatial stream networks have been previously applied to investigate biodiversity patterns in riparian insect communities^13^. Here, we have extended the application of SSN models to identify the ecological drivers that underlie population genetic diversity patterns within basins and quantify how the impacts of these drivers change across an environmental gradient. The integration of these models with increasingly available datasets that survey different community compositions based on environmental DNA can be used to map biodiversity hotspots and depict connectivity networks. This application would assist in decision making to prioritize the conservation of biodiversity hotspots and can be applied to draw mitigation and restoration measures to enhance gene flow among disconnected populations^1^. In addition, quantifying connectivity changes after adding or removing multiple barriers is a top concern of catchment planning^30^; thus, simulating alternative scenarios is a promising application of the stream-network approach for riverine species management. Simulation techniques can also be used to optimize sampling strategies for different purposes in stream networks or provide recommendations about sample sizes needed to achieve study objectives. These applications can significantly aid in the design of efficient monitoring strategies at relatively low costs^10^.

Overall, refining our capacity to describe, predict and simulate the amount and distribution of genetic diversity harboured by riparian populations of foundation species can improve adaptive management through cost-effective monitoring designs for conservation^31^. Preserving their potential for future adaptation will enhance the resilience of riparian population networks with cascading effects on associated biological communities, ecosystem functions and services, contributing to ecologically successful river management.

## Materials and Methods

### Field sampling

In the summers of 2010–2012, we conducted field surveys in riparian forests in 24 river valleys located in the Western Iberian Peninsula, across eight independent catchment systems: Tua-Douro, Zêzere-Tagus, Aljezur, Seixe, Odiáxere, Arade, Quarteira, and Guadiana (Fig. 2). These basins are spatially distributed within three regions: the Tua and Zêzere regions, which exactly match the Tua and Zêzere river basins, respectively, and the Algarve region, which includes six basins. The study area spans from the southernmost distribution of *S. salviifolia* and largely covers its latitudinal range and approximately one-third of its longitudinal range. We sampled 30 sites (each site representing a population) totalling 605 trees that were georeferenced with a submetre precision handheld GPS (Ashtech MobileMapper100). We sampled 15 or more individuals along the river reaches, and we collected as many individuals as possible (up to six) in low-density reaches to survey a similar sample area per site. We collected six healthy leaves per tree, and we stored them in paper bags containing silica gel until further work in the lab.

### Genotyping of biological samples

Genomic DNA was isolated from the dry leaf tissue per individual following standard methods (Supplementary Information S3). The samples were genotyped based on twelve polymorphic microsatellite markers; seven primers identified for *S. salviifolia*^32^ and five optimized from *S. burjatica* (cv. Germany)^33^ (Supplementary Information S3). We used samples from population 2 (N = 54) to test the performance of this set of polymorphic markers. Specifically, we examined the genetic corelation among loci (Supplementary Table S4A), tested for genotypic linkage disequilibrium (Supplementary Table S4B), and tested the ability of markers to discriminate among individuals (Supplementary Table S4C). Moreover, we estimated the expected number of individuals with the same genotype for an increasing number of loci (Supplementary Table S4D), estimated the probability of identical genotypes arising from sexual reproduction and random mating (Supplementary Table S4E), and estimated the incidence of null alleles and scoring error with a subset of N = 20 blind duplicated samples. Across loci, we detected low incidences of null alleles (2*10^−4^) and scoring errors (2*10^−3^) by applying Microchecker 2.2.3^34^. We also applied pedant 1.0^35^ to duplicated samples to estimate the per-allele maximum likelihood allelic dropout (ε_1_ = 0.2, CI = [0.00–0.5]) and false alleles (ε_2,_ CI = 0.1 [0.00–0.3]). Our study species exhibits clonal reproduction; therefore, we used the R package *Rclone*^36^ to identify identical genotypes, estimate the probability that they have been generated by independent sexual reproduction events, and evaluate the discriminative power of our 12 polymorphic markers to identify unique multilocus genotypes (Supplementary Tables S4F, S4G). We identified 5 clones out of 605 individuals.

### Environmental data

Based on previous studies^14^, we expected that spatial factors (Euclidean and dendritic spatial structures), bioclimatic variables (winter cold stress, summer drought stress), altitude and hydrology would determine the spatial distribution of the genetic variation of *S. salviifolia* at different spatial scales. We calculated environmental variables based on the GPS coordinates of the populations and individuals. We used two bioclimatological indices: the thermicity index (BIOC.TH) depicts the thermal envelope where plant species thrive, while the summer ombrothermic index (BIOC.SO) estimates the intensity of summer drought^37^. Geographic and hydrological variables were inferred from a digital elevation model (DEM) downloaded from the Shuttle Radar Topography Mission (SRTM) 90 m Digital Elevation Database v4.1^38^. We projected the Iberian Peninsula territory to the Lambert azimuthal equal-area projection, which guarantees equal pixel areas (indispensable for the hydrologic calculations), and simultaneously interpolated it to 35 m resolution. The altitude (ALT) at each site was extracted from the produced DEM. Then, to estimate the potential discharge, we derived a hydrographic network and computed a hydrologic index (DA) consisting of the drainage area of each site that was weighted by the total annual precipitation (P) in its contributing area. This hydrologic index distinguishes catchments presenting similar dimensions but occurring in regions with different P, providing a surrogate for total discharge.

### Spatial data

We generated the spatial data necessary for geostatistical modelling in ArcGIS 9.2^39^ using the Functional Linkage of Water Basins and Streams (FLoWS) and the Spatial Tools for the Analysis of River Systems (STARS) geoprocessing toolboxes. We applied the FLoWS toolset^40^ to construct a landscape network, which is a spatial data structure that stores the topological relationships between nodes (stream confluences) and directed edges (stream segments). For the analyses at the individual level, we incorporated the position of each sampled tree into the landscape network. For the analyses at the population level, we used the position of the central tree to map each site. Based on the landscape network, we used the STARS toolset to generate^41^ (1) hydrologic distances, (2) weights for converging stream tributaries thought to have stronger influences downstream, and (3) the SSN objects that contain feature geometry, attribute data, and topological relationships of the dataset, which are intended for geostatistical modelling within the *SSN* R package^42^.

### Estimates of genetic diversity and differentiation

To gain a comprehensive depiction of the genetic structure observed per study region, we calculated two types of estimators^43^: (i) fixation measures (F_st_, Phi_st_, G_st_, G’_st_); and (ii) allelic differentiation measures (D_Jost_). Given the controversy about the ability of F_*st*_ to quantify genetic structure^44,45^ when applying highly polymorphic genetic markers we opted for reporting four fixation measures (Supplementary Information S4) as implemented in GeneAlex 6.5^46^. We tested for Hardy-Weinberg equilibrium (HWE) and linkage disequilibrium (LD) at each site by using *GENEPOP*^47^ and, for multiple tests, we applied the B-Y method^48^, which is a modified Bonferroni method recommended for conservation genetics studies^49^. We estimated the following population genetic diversity metrics by applying the R package *gstudio*^50^: observed and expected unbiased heterozygosity (H_o_ and uH_e_, respectively), mean number of alleles (AR), effective number of alleles (A_e_), and mean number of private alleles (PA). We used INEST 2.0^51^ to estimate the F_is_ per population while considering the frequency of null alleles. We calculated the homozygosity level (HL) for each individual as implemented in the *adegenet* R package v.3.2.2.^52^. HL works as a proxy of individual inbreeding and it provides insights on mating patterns at the population level that are expected to change across an environmental gradient, with increased HL levels expected in small populations and those poorly connected by water flow, partly due to genetic drift. Different components of the genetic diversity respond to the impact of ecological factors at a different pace; thus, allelic diversity typically changes fast after an ecological perturbation, whereas uH_e_ and H_o_ show slow-paced changes^53^. Then, we evaluated the impacts of spatial and ecological factors on genetic diversity and structure by choosing a variety of estimators at the population level (A_e_, H_o_, uH_e_, F_is_) and the individual level (HL).

### Geostatistical modelling

We first performed an exploratory analysis to check for multicollinearity among environmental variables by inspecting the variance inflation factor (VIF). We retained all variables because they showed VIF values <2, suggesting no or little multicollinearity among study variables. We then modelled five genetic diversity estimators (A_e_, H_o_, uH_e_, F_is_, HL) with spatially explicit stream-network models by applying the R package *SSN*^42^.

Each SSN model accommodates a mixture of covariances that capture multiple spatial relationships in the dendritic network, including clustered measurements^10^. This method allows the data to determine the variance components that have the strongest influence rather than making an implicit assumption about the spatial structure^7^. Stream-network models accommodate two classes of autocovariance models that use hydrologic rather than Euclidean distance and are referred to as tail-down and tail-up models^7^. These models are based on a moving-average construction; so, spatial autocorrelation between sites occurs when their moving-average functions overlap (Fig. 1). A flow-connected spatial relationship results from water flowing from the upstream to the downstream location. A flow-unconnected relationship exists when two locations share a common junction downstream but are not connected by flow. In the tail-down models, the moving-average function (MAF) points in the downstream direction and therefore, spatial correlation is permitted between both flow-connected and flow-unconnected locations. In contrast, the MAF for the tail-up model points upstream, and therefore, spatial correlation is restricted to flow-connected locations (Fig. 1). A given SSN model is fitted using a mixed-covariance structure that combines two or more autocovariance models that may include the tail-up and tail-down autocovariance models and a traditional model based on Euclidean distance^7^.

We used a two-step model selection procedure as in Frieden *et al*.^13^ to select the model containing the most suitable covariance structure along with a set of environmental variables (covariates) that better explained the observed genetic patterns. First, we fixed the covariance structure and focused on covariate selection through an exhaustive screening of the candidate models that resulted from every linear combination of covariates. In this stage, we applied maximum likelihood to estimate model parameters, and we used Akaike’s information criterion for covariate selection, which prevents over-fitting of the model^54^. Then, we fixed the selected covariates and compared every linear combination of tail-up, tail-down and Euclidean covariance structures, testing four different autocovariance functions for each model type: the spherical, exponential, Mariah and linear-with-sill functions for tail-down and tail-up models; and the spherical, exponential, Gaussian and Cauchy functions for the Euclidean model as recommended by Peterson & Ver Hoef^7^. Overall, we tested 125 models (see Supplementary information S5 for details). For each response variable, we used restricted maximum likelihood^55^ with the root-mean-square-prediction error for the observations and the leave-one-out cross-validation predictions to select the final model^54^. Once we identified the final model for each response variable, we examined the influence of each variance component (tail-up, tail-down, Euclidean and nugget effect)^13^.

## Supplementary information

SUPPLEMENTARY INFORMATION

## Data Availability

Microsatellite genetic data are registered at GenBank (http://www.ncbi.nlm.nih.gov/genbank/), and the GenBank accession numbers are provided in Supplementary Information S3. Bioclimatic and hydrologic variables are available from http://home.isa.utl.pt/tmh/. The digital elevation model (SRTMv4.1) was downloaded from http://www.cgiar-csi.org/data/srtm-90m-digital-elevation-database-v4-1. STARS and FlOWS toolboxes for ArcGis, used to generate spatial data, were downloaded from “Tools for Spatial Statistical Modeling on Stream Networks” in the website https://www.fs.fed.us/rm/boise/AWAE/projects/.
