# Supplementary material for: A spatial stream-network approach assists in managing the remnant genetic diversity of riparian forests
Source: Sci Rep. 2019 May 1;9:6741. doi: 10.1038/s41598-019-43132-7 (PMC6494995; doi:10.1038/s41598-019-43132-7)
Supplement: Supplementary file 1 — SUPPLEMENTARY INFORMATION [file 41598_2019_43132_MOESM1_ESM.pdf]

SUPPLEMENTARY INFORMATION

TITLE: A spatial stream-network approach assists in managing the remnant genetic diversity of riparian forests

AUTHORS: Patricia María Rodríguez-González (*)[1], Cristina García [2,3], António Albuquerque[1,4], Tiago Monteiro-Henriques[5], Carla Faria[1], Joana B Guimarães[6], Diogo Mendonça[6], Fernanda Simões[6], Maria Teresa Ferreira[1], Ana Mendes[7], José Matos[6], Maria Helena Almeida[1]

AFFILIATIONS

[1]CEF - Centro de Estudos Florestais, Instituto Superior de Agronomia, Universidade de Lisboa, Edifício Azevedo Gomes, Tapada da Ajuda 1349-017, Lisboa, Portugal.

[2] Institute of Integrative Biology, Department of Evolution, Ecology, and Behaviour, University of Liverpool, Crown Street, Liverpool L69 7ZB United Kingdom

[3]Plant Biology, CIBIO/InBio, Centro de Investigação em Biodiversidade e Recursos Genéticos, Laboratório Associado, Universidade do Porto, Campus Agrário de Vairão, 4485-661, Vairão, Portugal.

[4]Ecofield, Monitorizações, Estudos e Projectos, LDA, Carcavelos, Portugal.

[5]Centro de Investigação e Tecnologias Agroambientais e Biológicas, Universidade de Trás-os-Montes e Alto Douro, Quinta de Prados, Apartado 1013, 5000-801, Vila Real, Portugal.

[6]INIAV - Instituto Nacional de Investigação Agrária e Veterinária, I.P., Unidade Estratégica de Biotecnologia e Recursos Genéticos, Portugal

[7]LabOr- Laboratório de Ornitologia, ICAAM - Instituto de Ciências Agrárias e Ambientais Mediterrânicas, Universidade de Évora, 7002-554, Évora, Portugal

TITLE

A spatial stream-network approach assists in managing the remnant genetic diversity of riparian forests

AUTHORS

Patricia María Rodríguez-González, Cristina García, António Albuquerque, Tiago Monteiro-Henriques, Carla Faria, Joana B Guimarães, Diogo Mendonça, Fernanda Simões, Maria Teresa Ferreira, Ana Mendes, José Matos, Maria Helena Almeida

Supplementary Table S1

Fst Matrix per population sorted by Region

Estimated with GenAlex 6.5

Peakall, R. and Smouse P.E. (2012) GenAlEx 6.5: genetic analysis in Excel. Population genetic software for teaching and research-an update.*Bioinformatics* 28, 2537-2539.

Pairwise Population Matrix of Fst Values for Total (different colours represent the three regions, and numbers correspond to sampled populations, same codes as in Figure 2)

	Pop1	Pop2	Pop3	Pop4	Pop5	Pop6	Pop7	Pop8	Pop9	Pop10	Pop11	Pop12	Pop13	Pop14	Pop15	Pop16	Pop17	Pop18	Pop19	Pop20	Pop21	Pop22	Pop23	Pop24	Pop25	Pop26	Pop27	Pop28	Pop29	Pop30	
Pop1	0.000	0.001	0.001	0.001	0.001	0.001	0.001	0.001																							Pop1
Pop2	0.031	0.000	0.001	0.001	0.001	0.001	0.001	0.001																							Pop2
Pop3	0.046	0.021	0.000	0.001	0.002	0.001	0.091	0.001																							Pop3
Pop4	0.033	0.016	0.029	0.000	0.001	0.001	0.001	0.001																							Pop4
Pop5	0.030	0.023	0.039	0.020	0.000	0.001	0.002	0.001																							Pop5
Pop6	0.042	0.023	0.036	0.027	0.031	0.000	0.001	0.001																							Pop6
Pop7	0.039	0.021	0.019	0.024	0.025	0.033	0.000	0.001																							Pop7
Pop8	0.079	0.053	0.042	0.050	0.072	0.056	0.046	0.000																							Pop8
Pop9									0.000	0.001	0.001	0.001	0.001	0.001	0.001	0.001															Pop9
Pop10									0.018	0.000	0.001	0.001	0.001	0.001	0.002	0.001															Pop10
Pop11									0.025	0.020	0.000	0.001	0.003	0.001	0.010	0.001															Pop11
Pop12									0.049	0.038	0.042	0.000	0.001	0.001	0.001	0.001															Pop12
Pop13									0.029	0.021	0.027	0.050	0.000	0.001	0.005	0.001															Pop13
Pop14									0.025	0.018	0.032	0.050	0.034	0.000	0.006	0.001															Pop14
Pop15									0.028	0.022	0.025	0.046	0.032	0.026	0.000	0.001															Pop15
Pop16									0.047	0.030	0.050	0.071	0.037	0.043	0.044	0.000															Pop16
Pop17																	0.000	0.007	0.001	0.032	0.003	0.001	0.002	0.001	0.001	0.003	0.001	0.017	0.004	0.001	Pop17
Pop18																	0.084	0.000	0.001	0.039	0.002	0.001	0.001	0.002	0.001	0.001	0.001	0.046	0.004	0.001	Pop18
Pop19																	0.068	0.072	0.000	0.001	0.001	0.001	0.001	0.001	0.001	0.001	0.001	0.001	0.001	0.001	Pop19
Pop20																	0.058	0.059	0.040	0.000	0.003	0.002	0.001	0.001	0.001	0.014	0.001	0.007	0.010	0.001	Pop20
Pop21																	0.066	0.061	0.050	0.048	0.000	0.004	0.001	0.001	0.001	0.003	0.001	0.004	0.001	0.001	Pop21
Pop22																	0.083	0.089	0.058	0.055	0.051	0.000	0.001	0.001	0.001	0.009	0.001	0.001	0.002	0.001	Pop22
Pop23																	0.073	0.076	0.061	0.049	0.049	0.065	0.000	0.001	0.001	0.001	0.001	0.001	0.001	0.001	Pop23
Pop24																	0.097	0.111	0.084	0.081	0.063	0.086	0.081	0.000	0.001	0.001	0.001	0.001	0.001	0.001	Pop24
Pop25																	0.097	0.089	0.083	0.066	0.068	0.082	0.082	0.066	0.000	0.001	0.001	0.001	0.001	0.001	Pop25
Pop26																	0.063	0.075	0.052	0.044	0.044	0.044	0.052	0.064	0.069	0.000	0.001	0.001	0.001	0.001	Pop26
Pop27																	0.086	0.081	0.072	0.059	0.050	0.073	0.082	0.072	0.076	0.060	0.000	0.001	0.001	0.001	Pop27
Pop28																	0.066	0.057	0.069	0.055	0.056	0.077	0.057	0.075	0.063	0.059	0.066	0.000	0.001	0.001	Pop28
Pop29																	0.098	0.079	0.091	0.057	0.082	0.082	0.084	0.117	0.105	0.076	0.095	0.095	0.000	0.001	Pop29
Pop30																	0.141	0.103	0.107	0.093	0.103	0.114	0.111	0.136	0.131	0.109	0.124	0.120	0.082	0.000	Pop30
	Pop1	Pop2	Pop3	Pop4	Pop5	Pop6	Pop7	Pop8	Pop9	Pop10	Pop11	Pop12	Pop13	Pop14	Pop15	Pop16	Pop17	Pop18	Pop19	Pop20	Pop21	Pop22	Pop23	Pop24	Pop25	Pop26	Pop27	Pop28	Pop29	Pop30	

SUPPLEMENTARY INFORMATION (Appendix S2)

Title: A spatial stream-network approach assists in managing the remnant genetic diversity of riparian forests, **Authors:** Patricia María Rodríguez-González, Cristina García, António Albuquerque, Tiago Monteiro-Henriques, Carla Faria, Joana B Guimarães, Diogo Mendonça, Fernanda Simões, Maria Teresa Ferreira, Ana Mendes, José Matos, Maria Helena Almeida

Appendix S2

Genetic diversity estimators and genetic differentiation estimates per region. This table reports the number of genotyped individuals (N), the number of sites per region (Sites), the mean ± std. value of five estimators of the genetic diversity (Ho, uHe, PA, AR, and A_e), and five estimators of genetic differentiation among sites (F_{st}, Phi_{st}, G_{st}, G'_{st}, D_{Jost}). Mean values are estimates across loci and across sites for each region. All genetic differentiation values resulted significantly different from zero ($p < 0.05$) based on a Z-test.

**Region**	**Genetic Diversity**							**Genetic Fixation and Differentiation**				
	N	Sites	H_o	uH_e	PA	AR	A_e	F_{st}	Φ_{st}	G_{st}	G'_{st}	D_{Jost}
**Tua**	214	8	0.74± 0.04	0.77±0.03	6	13.4±1.9	5.8±0.75	0.039	0.2035	0.0412	0.1806	0.1404
**Zezere**	212	8	0.75± 0.05	0.78±0.04	9	14.1±1.5	6.4±0.60	0.051	0.285	0.039	0.177	0.139
**Algarve**	179	14	0.72± 0.08	0.80±0.04	13	14.5±1.4	7.2±0.63	0.102	0.4812	0.0923	0.3558	0.2365

Ho: observed heterozygosity; uHe: unbiased expected heterozygosity; PA: total number of private alleles across sites per region; AR: mean allelic richness; Ae: mean effective number of alleles.

Statistics of genetic differentiation calculated among regions

Statistics of genetic differentiation: F_{st}, [1]; ii) Φ_{st}, [2]; iii) G_{st} [3]; iv) G'_{st} [4]; and v) D_{Jost} [5].

References

1 Weir, B. S. & Cockerham, C. C. Estimating F-Statistics for the Analysis of Population Structure. *Evolution* **38**, 1358-1370, doi:10.2307/2408641 (1984).
2 Meirmans, P. G. Using the AMOVA framework to estimate a standardized genetic differentiation measure. *Evolution* **60**, 2399-2402, doi:doi:10.1111/j.0014-3820.2006.tb01874.x (2006).
3 Nei, M. *Molecular evolutionary genetics*. (Columbia University Press, New York., 1987).
4 Hedrick, P. W. A standardized genetic differentiation measure. *Evolution* **59**, 1633-1638, doi:doi:10.1111/j.0014-3820.2005.tb01814.x (2005).
5 Jost, L. GST and its relatives do not measure differentiation. *Molecular Ecology* **17**, 4015-4026, doi:doi:10.1111/j.1365-294X.2008.03887.x (2008).

SUPPLEMENTARY INFORMATION (Appendix S3)

Title: A spatial stream-network approach assists in managing the remnant genetic diversity of riparian forests, **Authors:** Patricia María Rodríguez-González, Cristina García, António Albuquerque, Tiago Monteiro-Henriques, Carla Faria, Joana B Guimarães, Diogo Mendonça, Fernanda Simões, Maria Teresa Ferreira, Ana Mendes, José Matos, Maria Helena Almeida

Appendix S3. Genotyping of biological samples and characteristics of primers

Genotyping of biological samples

Genomic DNA was isolated from 50-100mg of dry leaf tissue per individual, cut in small pieces and placed into a 2 mL innuspeed Lysis Tube P (AnalyticJena, Germany). Leaf material was homogenized using a SpeedMill P12 (AnalyticJena, Germany). We used innuPREP Plant DNA Kit (AnalyticJena, Germany) to isolate genomic DNA following the manufacturer's instructions. Samples yielded between 30-100 ng/µL and they were genotyped based on twelve polymorphic microsatellite primers. Five loci were transferred from *S. burjatica* (cv. Germany)[1] and seven loci were previously identified for *S. salviifolia*[2]. All forward primers were fluorescently labelled using D3 or D4 labelling. PCR products were produced in a multiplex system using Qiagen PCR Multiplex kit instructions (see below primer mix composition). The following PCR cycle was used on a GeneAmp PCR System 2700 Thermal Cycler (Applied Biosystems, USA): activation at 95ºC 15 min; amplification (35 cycles) at 94 ºC for 30 s, 54 ºC for 90 s, and 72 ºC for 60 s; final extension at 72 ºC for 10 min. PCR products were analysed in a CEQ™ 8000 Beckman Coulter and allele determination was performed using the CEQ Genetic Analysis software.

Across loci, we detected a low incidence of null alleles ($2*10^{-4}$), and scoring errors ($2*10^{-3}$) by applying MICROCHECKER [3]. We used PEDANT 1.0 [4] and duplicated samples to estimate the per-allele maximum likelihood allelic dropout (ε_1 =0.2, CI= [0.00- 0.5]) and false alleles (ε_2, CI= 0.1 [0.00-0.3]). The multilocus genotyping showed that 5 individuals out of 605 were clones (0.82% of clones for the studied twelve loci).

Characteristics of twelve primers from Salix salviifolia.

Locus ID SB correspond to locus from *Salix burjatica* and Locus ID SS correspond to locus identified in *Salix salviifolia*. The table reports for each locus: *i)* the GenBank accession number;

ii) primer sequences (forward and reverse); *iii)* motif; *iv)* expected size; *v)* T_a, annealing temperature; *vi)* fluorescent dye added to the 5' end of the forward primer; *vii)* concentration of each primer in the primer premix; and *viii)* *A*, the number of scored alleles (for all individuals).

Locus ID	GenBank	Primer sequences	Motif	Size (pb)	T_a	*Dye*	A
**SB24**	AF442691	F: ACTTCAATCTCTCTGTATTCT<br>R:CTATTTATGGGTTGGTCGATC	$[TG]_{21}AG[TG]_3AG[TG]_3$	167	46	D4	21
**SB80**	AF442696	F: TAATGGAGTTCACAGTCCTCC<br>R: ATACAGAGCCCATTTCATCAC	$(tc)_{21}$	140	52	D3	13
**SB196**	AF442704	F: CTGTTTCCTGCCACTATTACC<br>R: TATAATCTGTCTCCTTTTGGC	$(gcc)_9$	184	47	D4	7
**SB199**	AF442705	F: CTATTTGGTCTCAATCACCTT<br>R: CTTTACCTCAGAAAATCCAGA	$[TG]_{11}CG[TG]_6$	122	50	D4	18
**SB194**	AF442703	F: TGTGAGATAAGATTTGTCGGT<br>R: CCATAAATAAAAAACGTGAAC	$(ca)_{14}$	122	50	D3	18
**SSDI3**	KJ670393	F: CAGTTTTCTGCTGCTGTTTCTG<br>R: GAAAGCCGAGGATCAAATCA	$(ag)_{10}$	176	51	D3	7
**SSDI4**	KJ670394	F:TCAAGAATTTGCACCACCAA<br>R: CTCCCCTCACACTCACACAC	$(gt)_{12}$	136	58	D4	25
**SSTRI2**	KJ670403	F: ATTTCCCCGTGTGACACATT<br>R: GGCTTTCAGACCCATACCCA	$(cat)_5ct(cat)_2$	186	53	D3	11
**SSTRI4**	KJ670405	F: CGCTTTCAATAAGGGGGTTT<br>R: TATGGCAGGGAACTGGGTAG	$(tat)_8$	212	55	D4	8
**SSTRI15**	KJ670414	CAAACTTGGCCGAGTGAGTT<br>GGCCTGCCAAATAAAAACAC	$(aat)_{13}$	184	52	D3	13
**SSTRI8**	KJ670409	F: CAAACTTGGCCGAGTGAGTT<br>R: GGCCTGCCAAATAAAAACAC	$(ttc)_8$	193	58	D4	11
**SSTRI10**	KJ670410	F: ATGCACCCAAACAAATTTCA<br>R: AGGGTTGTCGAAACATGGAG	$(ata)_{12}$	140	53	D3	9

Statistics of genetic differentiation calculated among regions

Statistics of genetic differentiation: F_{st}, [5]; ii) $\boldsymbol{\Phi}_{st}$, [6]; iii) G_{st} [7]; iv) G'_{st} [8]; and v) D_{Jost} [9].

References

1 Barker, J. H. A., Pahlich, A., Trybush, S., Edwards, K. J. & Karp, A. Microssatellite markers for diverse *Salix* species. *Molecular Ecology Notes* **3**, 4-6 (2003).

2 Simões, F. *et al.* in *Restauro fluvial e gestão ecológica: manual de boas práticas de gestão de rios e ribeiras* (eds J. Camprodon, M.T. Ferreira, & M. Ordeix) 338-345 (CTFC, 2012).

3 Oosterhouth, C. V., Hutchinson, W. F., Wills, D. P. M. & Shipley, P. MICRO-CHECKER: software for identifying and correcting genotyping errors in microsatellite data. *Molecular Ecology Notes* **4**, 535-538 (2004).

4 Johnson, P. C. D. & Haydon, D. T. Software for quantifying and simulating microsatellite genotyping error. *Bioinformatics and Biology Insights* **1**, 71-75 (2007).

5 Weir, B. S. & Cockerham, C. C. Estimating F-Statistics for the Analysis of Population Structure. *Evolution* **38**, 1358-1370, doi:10.2307/2408641 (1984).

6 Meirmans, P. G. Using the AMOVA framework to estimate a standardized genetic differentiation measure. *Evolution* **60**, 2399-2402, doi:doi:10.1111/j.0014-3820.2006.tb01874.x (2006).

7 Nei, M. *Molecular evolutionary genetics*. (Columbia University Press, New York., 1987).

8 Hedrick, P. W. A standardized genetic differentiation measure. *Evolution* **59**, 1633-1638, doi:doi:10.1111/j.0014-3820.2005.tb01814.x (2005).

9 Jost, L. GST and its relatives do not measure differentiation. *Molecular Ecology* **17**, 4015-4026, doi:doi:10.1111/j.1365-294X.2008.03887.x (2008).

SUPPLEMENTARY INFORMATION (Appendix S4)

Title: A spatial stream-network approach assists in managing the remnant genetic diversity of riparian forests, **Authors:** Patricia María Rodríguez-González, Cristina García, António Albuquerque, Tiago Monteiro-Henriques, Carla Faria, Joana B Guimarães, Diogo Mendonça, Fernanda Simões, Maria Teresa Ferreira, Ana Mendes, José Matos, Maria Helena Almeida

Appendix S4. Evaluation of the performance of a battery of 12 polymorphic SSRs markers

We used samples from population 2 (N=54) to test the performance of this battery of polymorphic markers. First, we examined the genetic correlation among loci applying a Chi-square test for Hardy-Weinberg Equilibrium as implemented in GenAlex 6.5 (Table S3A). We also tested for genotypic linkage disequilibrium (LD) for each pair of loci using the log likelihood ratio statistic as implemented in GENEPOP[11]. We applied a modified Bonferroni test referred as the B-Y method[2] to establish the threshold α-value for multiple comparisons (Table S3B). Then, we tested the ability of this set of markers to discriminate among individual genotypes by estimating the overall Probability of Identity (PI) and the PI for siblings as implemented in GenAlex (Table S3C). We also estimated the expected number of individuals with the same genotype for an increasing number of locus (Table S3D). We also estimated the probability of identical genotypes arising from sexual reproduction and random mating for all individuals sampled in population 2 as implemented in GenAlex 6.05[3]. (Table S3E). Finally we estimated the incidence of genotyping errors by genotyping a subset of N=20 blind samples that we used to estimate the incidence of null alleles and scoring error by applying MICROCHECKER[4]. Across loci, we detected a low incidence of null alleles ($2*10^{-4}$), and scoring errors ($2*10^{-3}$). We also applied PEDANT 1.0[5] [5]to duplicated samples to estimate the per-allele maximum likelihood allelic dropout (ε_1 =0.2, CI= [0.00-0.5]) and false alleles (ε_2, CI= 0.1 [0.00-0.3]). Our study species has clonal reproduction and therefore, we used the R package Rclone[6] to identify identical genotypes, to estimate the probability that they have been generated by independent sexual reproduction events and to evaluate the discriminative power of our 12 polymorphic markers to identify unique multilocus genotypes (Tables S3F, S3G). The multilocus genotyping identified 5 clones out of 605 individuals.

Table S4A We used the genotypes of individuals sampled (N=54) in one population (T2) to evaluate the performance of the set of 12 SSRs markers applied in this study. We examined the genetic correlation among loci applying a Chi-square test for Hardy-Weinberg Equilibrium as implemented in GenAlex 6.5. We detected departures from HWE at loci SSTRI8.

Locus	DF	ChiSq	Prob	Signif
**SB24**	120	137.962	0.125	ns
**SB80**	36	38.225	0.369	ns
**SB196**	10	5.815	0.831	ns
**SB199**	91	90.355	0.499	ns
**SB194**	91	88.329	0.560	ns
**SSDI3**	15	11.609	0.708	ns
**SSDI4**	136	104.011	0.981	ns
**SSTRI2**	36	33.125	0.606	ns
**SSTRI4**	21	23.261	0.330	ns
**SSTRI15**	55	66.974	0.129	ns
**SSTRI8**	55	94.635	0.001	***
**SSTRI10**	28	20.739	0.836	ns

Table S4B Results after test for genotypic linkage disequilibrium (LD) for each pair of loci using the log likelihood ratio statistic as implemented in GENEPOP[1]. We applied a modified Bonferroni test referred as the Benjamini and Yecuteli[2] method (B-Y method) to establish the threshold α-value for multiple comparisons. For 12 markers we obtained 66 pairwise p-values, which yields a critical α-value = 0.01047. Twelve p-values samples out of 66 below that threshold (highlight in bold).

Pop	Locus#1	Locus#2	P-Value
**T2-054**	SB80	SB196	0.834980
**T2-054**	SB196	SSTRI10	0.776420
**T2-054**	SSDI3	SSTRI8	0.684790
**T2-054**	SB194	SSDI3	0.664080
**T2-054**	SSTRI4	SSTRI10	0.628620
**T2-054**	SB196	SSDI3	0.562880
**T2-054**	SSDI3	SSTRI10	0.560680
**T2-054**	SB196	SSTRI15	0.530710
**T2-054**	SB199	SSDI3	0.473300
**T2-054**	SB196	SSTRI4	0.462530
**T2-054**	SB199	SSTRI8	0.419920
**T2-054**	SB196	SSDI4	0.382890
**T2-054**	SSTRI4	SSTRI15	0.363230
**T2-054**	SB194	SSTRI2	0.330000
**T2-054**	SSTRI15	SSTRI10	0.323970
**T2-054**	SSTRI8	SSTRI10	0.323820
**T2-054**	SB196	SB194	0.293590
**T2-054**	SSTRI2	SSTRI15	0.242570
**T2-054**	SB24	SSDI3	0.241110
**T2-054**	SSTRI4	SSTRI8	0.236420
**T2-054**	SSDI3	SSDI4	0.226190
**T2-054**	SSDI3	SSTRI4	0.225170
**T2-054**	SSTRI2	SSTRI4	0.195570

**T2-054**	SB196	SSTRI2	0.178380
**T2-054**	SSDI3	SSTRI15	0.155980
**T2-054**	SB80	SSDI3	0.155230
**T2-054**	SB80	SSTRI15	0.154520
**T2-054**	SB194	SSTRI4	0.134500
**T2-054**	SB194	SSTRI15	0.130600
**T2-054**	SB196	SSTRI8	0.129820
**T2-054**	SSTRI2	SSTRI8	0.124230
**T2-054**	SSDI3	SSTRI2	0.107540
**T2-054**	SB194	SSTRI10	0.096900
**T2-054**	SB194	SSDI4	0.091160
**T2-054**	SB80	SB194	0.083200
**T2-054**	SB24	SB80	0.07841
**T2-054**	SB24	SB196	0.075430
**T2-054**	SB199	SSDI4	0.070190
**T2-054**	SB24	SSTRI2	0.066490
**T2-054**	SB196	SB199	0.058240
**T2-054**	SB24	SSDI4	0.057690
**T2-054**	SB199	SSTRI2	0.051490
**T2-054**	SB194	SSTRI8	0.051210
**T2-054**	SB24	SSTRI8	0.047310
**T2-054**	SB24	SSTRI15	0.038800
**T2-054**	SB80	SSTRI4	0.031730
**T2-054**	SB199	SSTRI4	0.030110
**T2-054**	SSTRI2	SSTRI10	0.030000
**T2-054**	SSDI4	SSTRI2	0.026450
**T2-054**	SB80	SSDI4	0.024490
**T2-054**	SB24	SSTRI10	0.021240
**T2-054**	SB199	SSTRI10	0.013460
**T2-054**	SB199	SB194	0.012650

**T2-054**	SB80	SSTRI10	0.011640
**T2-054**	SB24	SSTRI4	**0.008160**
**T2-054**	SB24	SB199	**0.008040**
**T2-054**	SSDI4	SSTRI15	**0.003020**
**T2-054**	SB199	SSTRI15	**0.001010**
**T2-054**	SSTRI15	SSTRI8	**0.000940**
**T2-054**	SB80	SSTRI8	**0.000160**
**T2-054**	SB80	SSTRI2	**0.000080**
**T2-054**	SSDI4	SSTRI4	**0.000000**
**T2-054**	SSDI4	SSTRI8	**0.000000**
**T2-054**	SB80	SB199	**0.000000**
**T2-054**	SB24	SB194	**0.000000**
**T2-054**	SSDI4	SSTRI10	**0.000000**

Table S4C

Probability of identity at a locus (PI) and probability of identity for sibs at a locus (PI_{sibs}) tested for N=54 individuals. The PI is the average probability that two unrelated individuals drawn from the same randomly mating population will have the same multilocus genotype by chance. PI_{sibs} additionally takes into account the genetic similarity among individuals. Both are used as in molecular ecology as an indication of the minimum set of polymorphic markers required for reliable genetic tagging. Following Peakall & Smouse[3], the probability of identity at a locus is estimated as $2*[Sum(p_i^2)^2]-Sum(p_i)^4$, where p_i is the frequency of the ith allele at a locus and the probability of identity for sibs at a locus is estimated as $0.25+ [0.5* Sum(p_i^2)]+[0.5 * Sum(p_i^2)^2]-[0.25* Sum(p_i)^4]$. The expected number of individuals with the same genotype at a given locus based on PI and PI_{sibs}, respectively, is based on the population size (N=54). All estimates were obtained in GenAlex 6.5.

Locus	PI	Pi_{sibs}	Expected N Individuals with the same genotype at a given locus	Expected N individuals with the same genotype at a given locus based on PI_{sibs}
**SB24**	1.8E-02	3.0E-01	9.6E-01	1.6E+01
**SB80**	6.0E-02	3.6E-01	5.8E-02	5.9E+00
**SB196**	2.5E-01	5.2E-01	1.5E-02	3.1E+00
**SB199**	2.8E-02	3.2E-01	4.1E-04	9.9E-01
**SB194**	2.9E-02	3.2E-01	1.2E-05	3.2E-01
**SSDI3**	1.8E-01	4.7E-01	2.1E-06	1.5E-01
**SSDI4**	2.9E-02	3.2E-01	6.1E-08	4.7E-02
**SSTRI2**	8.3E-02	3.9E-01	5.1E-09	1.9E-02
**SSTRI4**	1.7E-01	4.8E-01	8.8E-10	8.9E-03
**SSTRI15**	6.2E-02	3.6E-01	5.5E-11	3.2E-03
**SSTRI8**	3.5E-02	3.3E-01	1.9E-12	1.1E-03
**SSTRI10**	1.2E-01	4.2E-01	2.3E-13	4.5E-04

Table S4D This table reports the expected number of individuals with the same genotype for increasing locus combinations. We report the probability of identity (PI) and the probability of identity among siblings (PI_{sibs}) for a population size N=54. PI and PI_{sibs} are estimated over multiple loci as the product of the individual locus PI's and PI_{sibs}'s.

Combination of loci	PI	PI_{sibs}	Expected N same genotype (PI)	Expected N same genotype (PI_{sibs})
1	1.8E-02	3.0E-01	9.6E-01	1.6E+01
1+2	1.1E-03	1.1E-01	5.8E-02	5.9E+00
1+2+3	2.7E-04	5.7E-02	1.5E-02	3.1E+00
1+2+3+4	7.6E-06	1.8E-02	4.1E-04	9.9E-01
1+2+3+4+5	2.2E-07	5.9E-03	1.2E-05	3.2E-01
1+2+3+4+5+6	3.9E-08	2.7E-03	2.1E-06	1.5E-01
1+2+3+4+5+6+7	1.1E-09	8.8E-04	6.1E-08	4.7E-02
1+2+3+4+5+6+7+8	9.4E-11	3.4E-04	5.1E-09	1.9E-02
1+2+3+4+5+6+7+8+9	1.6E-11	1.6E-04	8.8E-10	8.9E-03
1+2+3+4+5+6+7+8+9+10	1.0E-12	6.0E-05	5.5E-11	3.2E-03
1+2+3+4+5+6+7+8+9+10+11	3.5E-14	2.0E-05	1.9E-12	1.1E-03
1+2+3+4+5+6+7+8+9+10+11+12	4.3E-15	8.3E-06	2.3E-13	4.5E-04

Table S4E This table reports the probability of identical genotypes arising from sexual reproduction and random mating for all individuals sampled in population 2 (T2) as implemented in GenAlex 6.05[3].

ID	Multilocus genotype (12 SSRs)	Genotype probability
**T2-001**	164170109131174174109117105113177179135191189189212212183186202202132132	7.5E-16
**T2-002**	164170119129174180109117111125177179191203180186212218183186178184132138	4.9E-16
**T2-003**	170174119119174180117119125127177179161163180183203212180183187196135141	1.8E-15
**T2-004**	164189109119174180109135105109175177161203177180212215183186178184129141	9.6E-18
**T2-005**	162178107131180180117117109123173177135163180189212212180183181190132132	2.5E-19
**T2-006**	172189129129174180127131125127175175137205174186203212180183181181132132	1.6E-17
**T2-007**	174178119129171180107109125127179179133137177180203215183186187196132135	8.0E-17
**T2-008**	170187131135174180131137105109175175133163180186212212165174187196129141	7.7E-17
**T2-009**	164166119125174180109121105107175177135137180186215212183192181187129129	1.1E-16
**T2-010**	164176131131174174109119109109177177133161180183212212186189199205129132	2.4E-16
**T2-011**	176178129131174180133137109125175183133191183189203218165183199202129132	1.1E-19
**T2-012**	164174129133174174121127111131175181133163186189212215180189178184129129	4.3E-18
**T2-013**	166178129133171174113119107125177179133137180180212215171180187187129132	1.4E-16
**T2-014**	170187131131174174109119109117179179137191186192212221165183196199129132	6.1E-18
**T2-015**	150191109133174180123129107111177179133139180186206215180186199205132132	1.7E-22
**T2-016**	141170109125174174117137105107175175159161180189203212180183181187132141	1.8E-18
**T2-017**	166189131131174171121127105113175179135163180186203212180189187202129129	1.9E-15
**T2-018**	172181135135174180109121109131175179133205174189212212171186187193135138	2.2E-17
**T2-019**	164170131131174180117127105105179179161205180189212212165183178187129141	3.7E-14
**T2-020**	141189109131174180109109105125175179133161180180212218174183187202135141	1.2E-15
**T2-021**	164174119129174174109109113125177179205205180183215218171189187190132141	2.5E-17
**T2-022**	172174109131177180109121105109177179133163180180212212171183196196132135	1.5E-15
**T2-023**	168172131133174174111127113123177179137203186189203212177186187187120129	2.9E-19
**T2-024**	146172109129174180109121105109177179133137171180212212183186178196132135	9.2E-15
**T2-025**	146172109129174180109121105109179179133137171180212212183186178196132135	7.4E-15
**T2-026**	141146131131174180109121113125179179163205180180212212174183187202129129	2.5E-14
**T2-027**	172174129131174183129133107125177181133205180189203212177180193196129132	7.2E-19
**T2-028**	170189135135174180109127105125177179163205174180203218180186187202129129	4.9E-14
**T2-029**	170189135135174180109127105125177179163205174180203218183186187202129129	5.9E-14
**T2-030**	170189135135174180109127105125177179163205174180203218180186187205129129	2.9E-14
**T2-031**	170189135135171180109127105105175177163205174180203218180186187205129129	1.2E-15
**T2-032**	168170131135174174111119105127175179123133180180212212183201193208129129	2.2E-18
**T2-033**	162176129131180180109117113115179183135163177192212212180183187193129129	3.0E-17
**T2-034**	162189131135174180121121113125177183161169171186203212171183187205132132	4.9E-18
**T2-035**	174189125129171180127131105109177179191205174174212218171189193196132132	3.6E-18
**T2-036**	170170119131174174119127121125177177137163180180203212180183193193129129	4.0E-14
**T2-037**	162176129131174180117127105111179179133163174180212218171180187196129129	5.2E-14
**T2-038**	164187129131180180119121111133179179135163174174212212183192193193129132	1.7E-17
**T2-039**	162170131133174180111127109111175175137163174186212212183192193193135135	1.2E-17
**T2-040**	172172119131180180109119111113179179125133180180212218180186199202129129	3.7E-16

**T2-041**	164187129131174180121127121125179179163163174180212212180183187196132135	5.9E-14
**T2-042**	162170119131174174119119121127179179137161180180212212180183193193129135	8.1E-15
**T2-043**	162162129131174180117119121129179179137163180189212212183195184193129135	1.1E-16
**T2-044**	178191123129180180119119105109177177137169168177218221180183187190129129	1.3E-20
**T2-045**	146174119129171174121131109127179179137163180183203212180183181187120135	2.1E-17
**T2-046**	141170107129174174107107113129175177137161180192212215171180187202132138	2.8E-19
**T2-047**	162170119123174174107137113125177179137205168180200212180186178184132132	1.2E-18
**T2-048**	162170131135171180119127107111175179163177189192212212171189187196129129	5.0E-17
**T2-049**	164164129131174174121133105111177177127135180180215221171189187190129141	2.6E-19
**T2-050**	170174119131174180109127109131179179125133171186212215180183178178138141	1.1E-17
**T2-051**	141172129129174174123127107113177179191211180186212218180183178178120132	1.7E-18
**T2-052**	181191129135171174119137115125177179137203180192203218177180184187123129	1.0E-20
**T2-053**	170178119125171180121123105107177179131163189189212215180183184196126132	1.3E-19
**T2-054**	162170131133171180107129109131179179137161180189212215180183187205129135	1.5E-16

Table S4F Results after computing main genotypic diversity and richness indices. The table includes the population code in the first column, the number of genotypes sampled (N), the number of unique genotypes identified (G), the clonal diversity index R estimated as G/N [7] as implemented in the *Rclone* R package.

1	N	G	R	F_{is}
Population\ Overall	605	600	0.991	0.011
1	18	18	1	0.0534
2	54	54	1	-0.007
3	18	17	0.94	-0.021
4	52	52	1	0.067
5	18	18	1	0.049
6	20	20	1	0.018
7	19	17	0.89	-0.026
8	15	15	1	-0.210
9	22	22	1	-0.039
10	54	54	1	-0.075
11	18	18	1	-0.029
12	55	55	1	-0.085
13	15	14	0.933	-0.038
14	18	18	1	-0.017
15	15	15	1	0.046
16	15	15	1	-0.029
17	6	6	1	0.115
18	6	6	1	0.101
19	30	30	1	-0.012
20	10	10	1	0.297
21	10	10	1	-0.065
22	10	10	1	0.047
23	20	20	1	0.045
24	10	10	1	0.058
25	26	26	1	0.079
26	10	10	1	0.048
27	14	14	1	-0.173
28	10	10	1	0.182
29	8	8	1	0.042
30	9	9	1	-0.072

Table S4G Probability that repeated genotypes originate from distinct sexual events computed with Round-Robin method.

Genotype 1	Genotype 2	Prob
**Individual #258**	Individual #257	4.6^{-14}
**Individual #362**	Individual #257	1.05^{-27}
**Individual #363**	Individual #257	1.60^{-41}
**Individual #372**	Individual #364	1.57^{-15}
**Individual #548**	Individual #543	1.45^{-14}

REFERENCES

1 Rousset, F. GENEPOP'007: a complete re-implementation of the GENEPOP software for Windows and Linux. *Molecular Ecology Resources* **8**, 103-106 (2008).

2 Benjamini, Y. & Yekutieli, D. The Control of the False Discovery Rate in Multiple Testing under Dependency. *The Annals of Statistics* **29**, 1165-1188 (2001).

3 Peakall, R. & Smouse, P. E. GenAlEx 6.5: genetic analysis in Excel. Population genetic software for teaching and research—an update. *Bioinformatics* **28**, 2537-2539, doi:10.1093/bioinformatics/bts460 (2012).

4 Oosterhouth, C. V., Hutchinson, W. F., Wills, D. P. M. & Shipley, P. MICRO-CHECKER: software for identifying and correcting genotyping errors in microsatellite data. *Molecular Ecology Notes* **4**, 535-538 (2004).

5 Johnson, P. C. D. & Haydon, D. T. Software for quantifying and simulating microsatellite genotyping error. *Bioinformatics and Biology Insights* **1**, 71-75 (2007).

6 Arnaud-Haond, S. & D., B. RClone: Partially clonal population analysis. R package version 1.0.2. https://CRAN.R-project.org/package=RClone. (2016).

7 Dorken, M. E. & Eckert, C. G. Severely reduced sexual reproduction in northern populations of a clonal plant, Decodonverticillatus (Lythraceae). *Journal of Ecology* **89**, 339-350, doi:doi:10.1046/j.1365-2745.2001.00558.x (2001).

SUPPLEMENTARY INFORMATION (Appendix S5)

Title: A spatial stream-network approach assists in managing the remnant genetic diversity of riparian forests, **Authors:** Patricia María Rodríguez-González, Cristina García, António Albuquerque, Tiago Monteiro-Henriques, Carla Faria, Joana B Guimarães, Diogo Mendonça, Fernanda Simões, Maria Teresa Ferreira, Ana Mendes, José Matos, Maria Helena Almeida

Appendix S5 –SSN Models tested and best models residuals

Table S5A. List of variables combinations for all models tested in the two-step selection modelling procedure. Response stands for the Response variable at the population (Ae, Number of effective alleles; Fis, Fixation index; Ho, observed heterozygosity); and at the individual level (HL, homozygotes level). The covariates (explanatory variables) tested include: Altitude (ALT) a hydrologic index consisting of the drainage area of each site weighted by total annual precipitation (DA), the thermicity index (BIOC.TH) and the summer ombrothermic index (BIOC.SO)[1]. We used a two-step model selection procedure as in Frieden *et al.*[2], to select the model containing the most suitable covariance structure along with a set of environmental variables (covariates) that better explained the observed genetic patterns. First, we fixed the covariance structure and focused on the covariates selection through an exhaustive screening of the candidate models resulting from every linear combination of covariates. In this stage, we applied maximum likelihood to estimate model parameters so that we used the Akaike's Information Criterion for covariate selection, which prevent over-fitting the model[3]. Then, we fixed the selected covariates and compared every linear combination of Tail-up, Tail-down and Euclidean covariance structures, testing four different autocovariance functions for each model type: the Spherical, Exponential, Mariah and Linear-with-sill function, for Tail-down and Tail-up models; and the Spherical, Exponential, Gaussian and Cauchy functions for the Euclidean model as recommended by Peterson & Ver Hoef[4]. Overall, we tested 125 models. For each response variable, we used restricted maximum likelihood[5] with the root-mean-square-prediction error for the observations and the leave-one-out cross-validation predictions to select the final model[3]. Once we identified the final model for each response variable, we examined the influence of each variance component (Tail-up, Tail-down, Euclidean and nugget effect)[2].

Response	1st step model selection	2nd step
Ae	**Covariates (all linear combinations)**	**Covariates (fixed)**
	DA+ALT+BIOC.SO+BIOC.TH	DA+ALT
	**Autocovariance functions (fixed)**	**Autocovariance functions (all linear combinations)**
	TU+TD+EUC	TU+TD+EUC
	TU: Mariah	TU: Spherical, Exponential, Mariah and Linear-with-sill function
	TD: Linear-with-sill	TD: Spherical, Exponential, Mariah and Linear-with-sill function
	EUC: Exponential	EUC: Spherical, Exponential, Gaussian and Cauchy
Fis	**Covariates (all linear combinations)**	**Covariates (fixed)**
	DA+ALT+BIOC.SO+BIOC.TH	DA+BIOC.TH
	**Autocovariance functions (fixed)**	**Autocovariance functions (all linear combinations)**
	TU+TD+EUC	TU+TD+EUC
	TU: Mariah	TU: Spherical, Exponential, Mariah and Linear-with-sill function
	TD: Linear-with-sill	TD: Spherical, Exponential, Mariah and Linear-with-sill function
	EUC: Exponential	EUC: Spherical, Exponential, Gaussian and Cauchy
uHe	**Covariates (all linear combinations)**	**Covariates (fixed)**
	DA+ALT+BIOC.SO+BIOC.TH	DA
	**Autocovariance functions (fixed)**	**Autocovariance functions (all linear combinations)**
	TU+TD+EUC	TU+TD+EUC
	TU: Mariah	TU: Spherical, Exponential, Mariah and Linear-with-sill function
	TD: Linear-with-sill	TD: Spherical, Exponential, Mariah and Linear-with-sill function
	EUC: Exponential	EUC: Spherical, Exponential, Gaussian and Cauchy

Ho	**Covariates (all linear combinations)**	**Covariates (fixed)**
	DA+ALT+BIOC.SO+BIOC.TH	DA+ALT
	**Autocovariance functions (fixed)**	**Autocovariance functions (all linear combinations)**
	TU+TD+EUC	TU+TD+EUC
	TU: Mariah	TU: Spherical, Exponential, Mariah and Linear-with-sill function
	TD: Linear-with-sill	TD: Spherical, Exponential, Mariah and Linear-with-sill function
	EUC: Exponential	EUC: Spherical, Exponential, Gaussian and Cauchy
HL	**Covariates (all linear combinations)**	**Covariates (fixed)**
	DA+ALT+BIOC.SO+BIOC.TH	BIOC.SO
	**Autocovariance functions (fixed)**	**Autocovariance functions (all linear combinations)**
	TU+TD+EUC	TU+TD+EUC
	TU: Mariah	TU: Spherical, Exponential, Mariah and Linear-with-sill function
	TD: Linear-with-sill	TD: Spherical, Exponential, Mariah and Linear-with-sill function
	EUC: Exponential	EUC: Spherical, Exponential, Gaussian and Cauchy

Table S5B. Residuals graphs for best models retained for each response variable at the population (Ae, Number of effective alleles; Fis, Fixation index; Ho, observed heterozygosity); and at the individual level (HL, homozygotes level). Histogram of standardized residuals and scatterplot of CV predictions vs standardized residuals.

Best model residuals	
Response variable at the population level	
Ae ~ ALT + DA + ("Spherical.tailup", "LinearSill.taildown")	
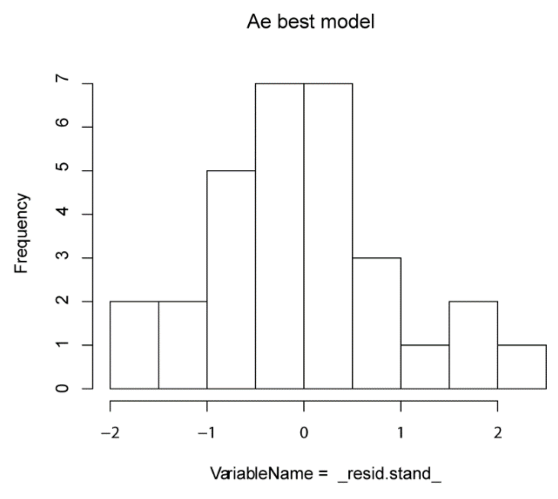	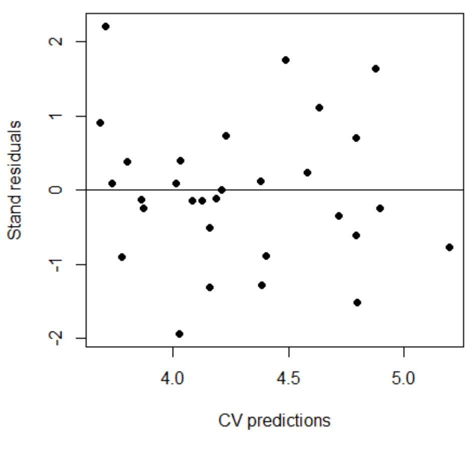
Fis ~ BIOC.TH + DA + ("Mariah.tailup", "LinearSill.taildown", "Exponential.Euclid")	
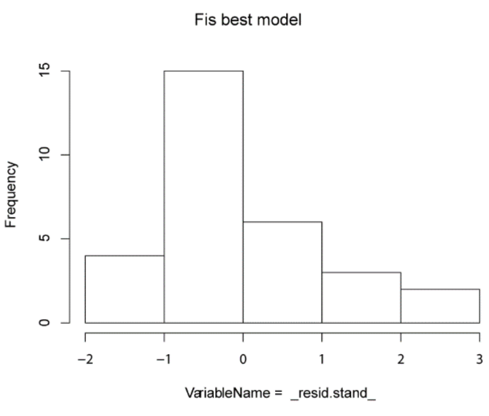	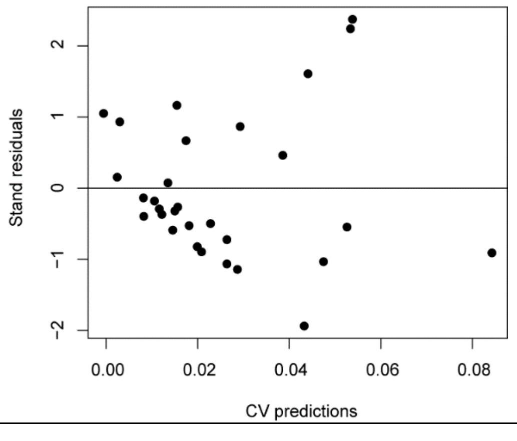

uHe~DA+ (“Exponential.tailup”, “Exponential.taildown”, « Gaussian.Euclid”)	
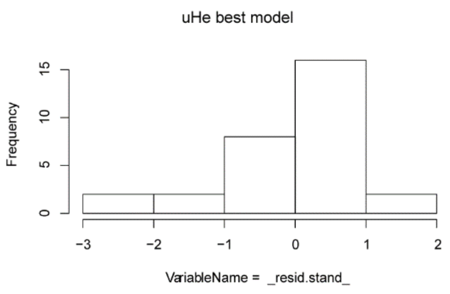	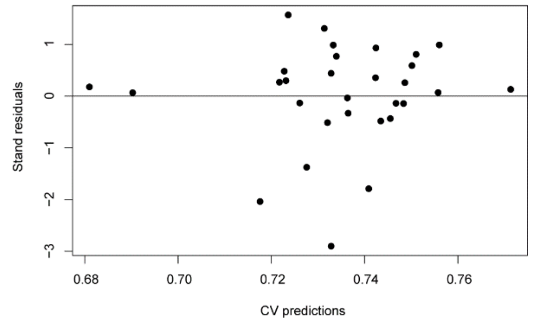
Ho~ALT+DA+ ("LinearSill.taildown", "Exponential.Euclid")	
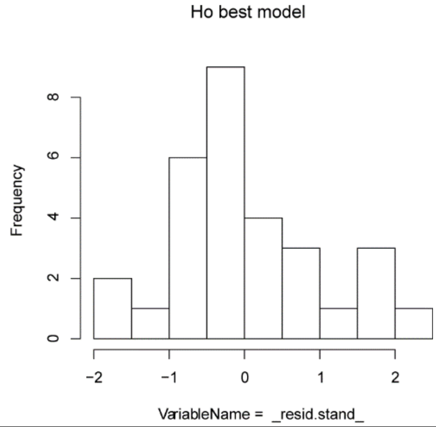	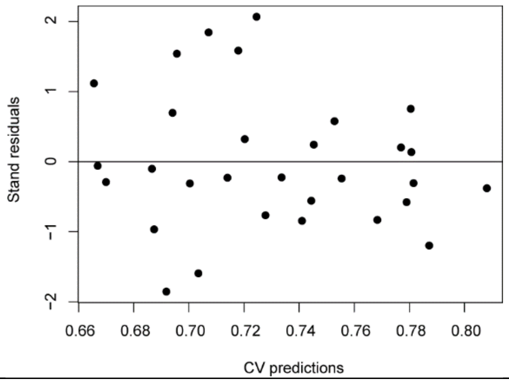
Response variable at the individual level	
HL~BIOC.SO+ (“Exponential.tailup”, “LinearSill.taildown",	
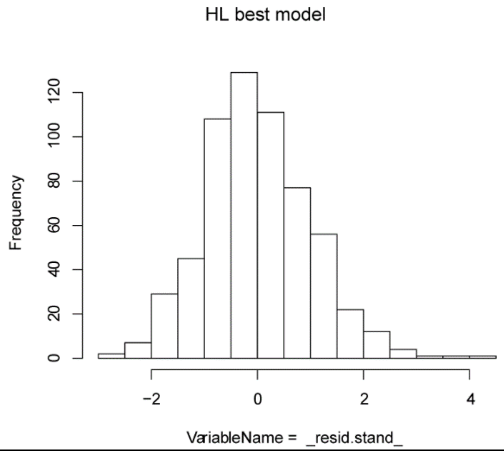	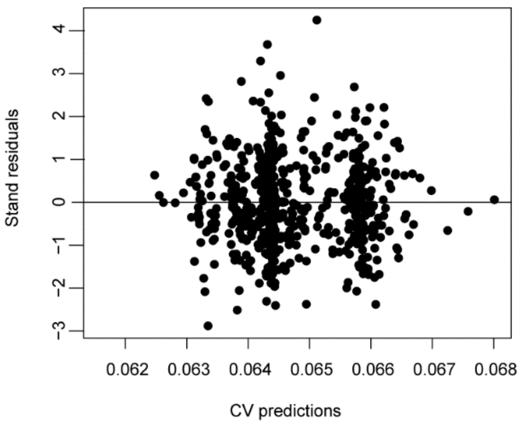

REFERENCES

1 Monteiro-Henriques, T. *et al.* Bioclimatological mapping tackling uncertainty propagation: application to mainland Portugal. *International Journal of Climatology* **36(1)**, 400-411, doi:10.1002/joc.4357 (2016).

2 Frieden, J. C., Peterson, E. E., Angus Webb, J. & Negus, P. M. Improving the predictive power of spatial statistical models of stream macroinvertebrates using weighted autocovariance functions. *Environmental Modelling and Software* **60**, 320-330 (2014).

3 Bennett, N. D. *et al.* Characterising performance of environmental models. *Environmental Modelling & Software* **40**, 1-20, doi:http://dx.doi.org/10.1016/j.envsoft.2012.09.011 (2013).
4 Peterson, E. E. & Ver Hoef, J. M. A mixed-model moving-average approach to geostatistical modeling in stream networks. *Ecology* **91**, 644-651 (2010).
5 Cressie, N. *Statistics for Spatial data (revised edition).* (Wiley, 1993).
